# How Do People With Intellectual Disability Engage With and Understand Gambling? A Qualitative Study of Adults in Victoria, Australia

**DOI:** 10.3389/fpubh.2020.536520

**Published:** 2021-01-12

**Authors:** Hannah Pitt, Samantha L. Thomas, Joanne Watson, Russell Shuttleworth, Kevin Murfitt, Susan Balandin

**Affiliations:** ^1^Faculty of Health, School of Health and Social Development, Institute for Health Transformation, Deakin University, Geelong, VIC, Australia; ^2^Department of Anthropology, Goldsmiths, University of London, London, United Kingdom

**Keywords:** gambling, poker machines, electronic gambling machines, gambling harm, responsible gambling, intellectual disabilities (ID)

## Abstract

**Objective:** This study aimed to understand the factors that may influence how and why people with intellectual disability may engage in gambling.

**Method:** Nineteen people with intellectual disability were recruited from a disability advocacy organization and participated in face to face, semi-structured qualitative interviews. Open ended questions were used to explore participants' gambling participation, recall of, and attitudes toward, different gambling products, understanding of gambling harm, and awareness of responsible gambling messages.

**Results:** All participants could remember gambling in their lifetime and some participants had recently engaged in gambling. Many participants were aware of different gambling products, and a few participants could describe in detail the technical aspects of electronic gambling machines. Most participants did not specifically recall seeing gambling harm minimization messages, however some described engaging in individual responsibility measures, such as limits and control, as they perceived this reduced the risks of experiencing harm.

**Conclusions:** People with intellectual disability are engaging with gambling products in a similar way to the general community. Therefore, it is important to understand the different pathways that may lead people with intellectual disability to initiate and continue gambling and to ensure that they are aware of and protected from the potential risk.

**Implications for Public Health:** Policy makers and practitioners should seek to understand and implement a range of strategies to reduce and prevent the harms associated with particular gambling products and environments for this population sub-group.

## Introduction

### Overview

Little is known about the gambling experiences of people with intellectual disability. Schalock and colleagues have recently provided an operational definition of intellectual disability describing it “*as a diagnosis or label given to individuals who meet the criteria of significant limitations both in intellectual functioning and adaptive behavior as expressed in conceptual, social, and practical skills, and is manifest before age 18*” [(1), p. 225]. Specifically, a person with an intellectual disability may have difficulties with adaptive functioning, including conceptual processing, social skills, communication and independent decision-making potentially placing them at greater risk of gambling harm. With the emergence of the social model of disability and more recently the United Nations Convention on the Rights of Persons with Disabilities (UNCRPD) (2), intellectual disability is increasingly seen through an ecological lens, whereby a person's disability is viewed as an interaction between themselves and the environment in which they live, work and play. When viewed in this way, and in line with Article 19 of the UNCRPD, there is a clear role for society in removing the disabling barriers and restrictions present in societal structures, that preclude a person with intellectual disability's ability to engage meaningfully within communities of their choice (3). As for all citizens, such meaningful community engagement is enhanced when a person has equal access to public health resources, supports and information, including evidence-based messaging relating to gambling harm.

Gambling harm is defined as “*any initial or exacerbated adverse consequence due to an engagement with gambling that leads to a decrement to the health or well-being of an individual, family unit, community or population*” [(4), p. 4]. This harm comprises negative financial impacts, health effects including mental health and stress issues, relationship breakdown, and crime (4). While problem gambling screening tools identify that about 1% of the population experience problem/pathological gambling, many others may be at low to moderate risk of experiencing some level of gambling harm (5). Gambling harm also extends beyond the individual gambler, with research indicating that for every person classified as having a problem with gambling up to 10 other people are negatively impacted (6, 7).

While gambling has traditionally been investigated through individual psychological and addiction treatment models (8), studies using a public health lens have focused on the role that socio-cultural, environmental, commercial (or industry), and political factors play in gambling related harm (9–11). This includes understanding how and why some population sub-groups, such as older adults, Indigenous Australians, culturally and linguistically diverse groups, children, and young men, may be particularly at risk of gambling harm (12–18). Despite this, very few studies have examined whether people with intellectual disability may also be a group at risk of experiencing harm from gambling.

### People With Intellectual Disability and Gambling

The research exploring the experiences of people with disability and gambling is limited to studies focused on young people with attention deficit and hyperactive disorder (ADHD) and learning difficulties (as opposed to intellectual disability). As is typical of general gambling research to date (19), the research has centered on individual characteristics and personality traits (20–22), rather than the influence of broader socio-cultural, environmental, and commercial determinants of gambling attitudes and behaviors. These findings were predominately quantitative and identified an association between disability and gambling risk using a range of screens that test for decision making and impulsivity (22, 23). Scheidemantel and colleagues also highlighted the lack of research that is in this area, and presented case reports from people with intellectual disability in psychiatric clinics and their experience of problem gambling (24). These reports articulate the complexities within this group including individuals spending large amounts of money on gambling and being resistant to group therapy treatment for their gambling.

While literature is limited there is a clear overlap between some of the common attributes experienced by people with intellectual disability and people who experience harm from gambling, creating concern that people with disability may be subject to the same risk of gambling harm but may not be aware of the risk or indeed supports available. Evidence from the Household Income, Labor and Dynamic Survey found that people classified as being at risk of problem gambling had lower levels of employment and received welfare payments (5). Other research has found that people living in lower socio economic areas, those who experience social problems and those who have limited decision making skills are also linked with experiencing gambling related harm (25, 26). Risk factors for developing gambling problems have also been identified as reduced social capital, loneliness, and social isolation (27–29). Given that social isolation, poverty, and reduced social capital have also been highlighted as a concern for people with intellectual disability (30, 31), it is important to understand how people with intellectual disability may be affected by gambling or be drawn into gambling environments.

### Influences on the Gambling Attitudes and Behaviors of People With Intellectual Disability

The normalization of gambling is a developing area of research that builds on the principles of a public health approach. Thomas and colleagues (10) defined the normalization of gambling as:

*The interplay of socio-cultural, environmental, commercial and political processes which influence how different gambling activities and products are made available and accessible, encourage recent and regular use, and become an accepted part of life for individuals, their families and communities* [(10), p. 54].

Using this approach, it is important to identify factors that may influence the attitudes of people with intellectual disability toward gambling. Recent research has focused on the interplay of environmental and commercial factors by exploring the role of community gambling venues in normalizing gambling amongst different population sub-groups (32, 33). In Australia, the most common community gambling venues are hotels (known as pubs) and clubs. Clubs are not for profit member based organizations that include bowling clubs, sporting and recreation clubs, golf clubs, Returned Servicemen's Leagues (RSLs), community and workers club, and cultural and religious clubs (34). Gambling products available in these venues can include electronic gambling machines (EGMs, pokies, or slots), sport and horse wagering, raffles, and bingo. People with intellectual disability, like many in the community, may attend venues where gambling occurs. These venues, in particular clubs, contain a range of non-gambling activities that may be particularly appealing and inclusive for people with intellectual disabilities and their families. These include all abilities leisure activities, entertainment, and cheap meals. However, research also shows that the more regularly people attend community gambling venues for non-gambling activities, the more likely they are to gamble at these venues, and the more likely they are to experience gambling related harm (32). Participants in a study by Pitt and colleagues, which explored the role of pubs and clubs in the lives of people with intellectual disability, reported that they attended these venues for the affordable meals and to socialize with family and friends (35). The socio-cultural influence of family and peers has also been explored with other at-risk population groups such as children. This research has found that the gambling traditions and behaviors that young people see their parents engaged with can often influence their own attitudes toward gambling (36). Given that social networks including family, friends and supporters play an important role in the lives of people with intellectual disability (31, 37), understanding how this normalizes gambling will also be important in future research.

The following study aims to understand the factors that may influence how and why people with intellectual disabilities may engage in gambling and if they differ from other groups in the community. The paper is guided by four research questions:

1. How do people with intellectual disability engage in gambling?
2. What is their knowledge of the structural characteristics of gambling products?
3. How do they conceptualize the harms associated with gambling and gambling products?
4. What are their perceptions and awareness of responsible gambling messages?

## Method

### Approach

This exploratory study focused on the gambling attitudes, behaviors, and knowledge of people with intellectual disability. It was part of a broader study that explored the role of pubs and clubs in the lives of people with intellectual disability (35). An advisory group was developed through contacts with a disability organization and a group of people with intellectual disability were included to discuss the initial design and development of the study. The interview question guide was piloted with this group to ensure questions were comprehendible and common language for gambling terms was used. The advisory group confirmed that the questions and topics explored were relevant to people with intellectual disability. Ethical approval was received from the Deakin University Human Research Ethics Committee [2018-140].

### Sampling and Recruitment

People with intellectual disability who could provide informed consent, participate in a face to face interview, aged 18 years and over, and who had attended a pub or club in the past 12 months were invited to participate in an interview. The research team worked with a local advocacy organization to identify potential participants. The advocacy organization recruited six interested members with intellectual disability to form an advisory group, hosted the meetings, and facilitated recruitment for the study. All participants, including those who provided advice to the project received a $50 gift voucher as reimbursement for their time.

The advisory group met with members of the research team for an initial consultation around the aims of the study, and to understand if it would be worthwhile research to conduct. This group also consulted with the researchers about the appropriateness and intelligibility of interview questions and identified the gambling terms that they considered were most commonly used. For example, the group advised the use of the term “pokies” instead of “EGMs.” The advocacy organization assigned two staff members to the project and the researchers provided them with the plain language statement. The staff members were responsible for identifying potential participants. Members of the research team were available to answer questions. Once a participant had consented to participate, a mutually convenient time to meet with a research team member for the interview was arranged.

### Data Collection

Data collection occurred during July and September 2018, with all interviews conducted face to face by authors one, two and three. Interviews were audio recorded with the permission of each participant. Interviews varied in length (from 10 min to 40 min). While it was important to interview individuals separately, the research design allowed for two participants to be interviewed together at their request. This ensured that the research was both inclusive and met the participant requirements and that participants in the study felt comfortable.

The analysis focused on the four research questions in the interview question guide that specifically related to gambling. This guide can be obtained from the first author. Questions included the participant's definitions of gambling, awareness and understanding of gambling products, conceptualization of risk and benefits of gambling, and interpretation of responsible gambling messages. The semi structured interviews were facilitated by using two picture boards. One picture board contained pictures of gambling and non-gambling activities within pubs and clubs and the second consisted of gambling products. Gambling activities and products included EGMs, sports betting, horse betting, casino games, bingo, raffles, and lotteries. Each product was also presented on a single A4 piece of paper to make it easier for participants to identify and discuss. The researcher made a subjective decision during each interview if any or all of these techniques would be effective in facilitating the interview. This methodological technique has been employed effectively in gambling research amongst young people to encourage their engagement and discussion within interviews (33, 38). Visual supports have also been used to support comprehension for people with intellectual disability in other areas of research (39–41).

### Data Analysis

Interviews were transcribed by a professional transcription company and uploaded to QSR NVivo 12. Braun and Clarke's thematic analysis was conducted, this uses a six step process (42). The key phases of analysis adapted from the six-step process are presented in Table 1. The analysis process included reading and re-reading the transcripts to ensure that the researchers were aware of the content of the interviews and that they had familiarized themselves with the data. The first author then coded the transcripts to identify the similarities, differences and the major points of discussion within each participant's responses. Participants' discussed the different product preferences, why they engaged with gambling products and what harm reduction strategies they thought would be important. The codes were compared within and across the interviews and grouped into themes as described in Table 1. Another researcher independently coded some of the transcripts and the codes were compared—any disagreements were resolved by consensus. Themes were then reviewed by the first three authors to ensure they were capturing meanings relevant to the research questions. All members of the research team came together to discuss their thoughts about the data and to compare and contrast common themes. Finally, the main themes were discussed with the advocacy organization to ensure that they had an opportunity to comment on the research.

**Table 1:** Thematic analysis process.

**Phase of analysis**	**Description of process**
Familiarization with data	During this process the transcripts were read and re read. This involved taking initial notes down about the different interviews.
Generating initial codes	Development of initial codes and collation of data relevant to each code in NVivo. For example: - Gambling product mentioned - Who they gambled with - Where they gambled - Attitudes about gambling: fun, exciting, harmful, risky - Knowledge about gambling products: specific features, sounds - Negative outcomes of gambling: financial, social, health - Seeking help strategies: Talking, professional help - Responsible gambling language: settling limits, control - Addiction
Searching for themes	Collating codes into potential themes, gathering all data relevant to each potential theme. For example: - Gambling behaviors - Gambling harm - Harm reduction strategies
Reviewing themes	Checking the themes work in relation to the coded extracts (Level 1) and the entire data set (Level 2).
Defining and naming themes	Ongoing analysis to refine the specifics of each theme, and the overall story the analysis tells, generating clear definitions and names for each theme. For example: - People with intellectual disability's engagement with gambling products - Conceptualization of gambling harm - Harm reduction and help seeking strategies
Producing the report	The final opportunity for analysis. Selection of vivid, compelling extract examples, final analysis of selected extracts, relating back of the analysis to the research question and literature, producing a scholarly report of the analysis.

## Results

### Participants

There were 19 participants and 18 interviews conducted in this study. Just over half of the sample were male (*n* = 10, 52.6%). The age of participants ranged from 20 to 70 years old with an average age of 44 years. Table 2 contains details about participants including a pseudonym created using a random name generator, participants gender, along with characteristics about their gambling venue engagement and gambling engagement.

**Table 2:** Participant characteristics and overview of venue and gambling experiences.

**Participant**	**Venue**	**Gambling engagement**
Deborah -Female	Attended RSL's about once a month. Went on her own for meals.	Had used EGMs but experienced harm so reported not going to that particular venue anymore. Reported gambling on raffles, no indication of timeframe.
Christopher -Male	Attended a pub. Went on his own to gamble and for meals. Reported having a member's card.	Currently gambled on EGMs.
Leah -Female	Attended a hotel about once a week, on the weekend and went with friends. Was a member and reported going to different venues for different reasons, such as food.	Currently gambled on EGMs when going to the pub with friends. Also gambled on Keno but not as often.
Richard -Male	Reported going to pubs weekly.	Had a range of gambling product knowledge: EGMs, keno, sports betting. But did not talk about currently gambling. Had gambled on the Melbourne Cup with family.
Nathan -Male	Attended RSL's with his family. Reported going for food and special occasions.	Didn't talk specifically about gambling but had knowledge of the TAB and EGMs. Said he had played the EGMs a long time ago and had used scratchies.
Alexandria -Female	Attended pubs, also worked at pubs. Had gone to venues with EGMs with her family.	Watched her family gamble on EGMs but did not currently gamble. Had been involved in Melbourne Cup sweeps with family.
Kyle -Male	Attended RSL's often on his own but sometimes with friends. Reported that he would go every day. Was a member and went to gamble and drink alcohol.	Reported gambling currently and regularly on EGMs and horses.
Kieron -Male	Went to an RSL with friends. Reported going once a fortnight, to gamble.	Reported regularly and currently gambling on EGMs.
Leonard -Male	Went to a pub close to his house. Would attend by himself. He reported many venue membership cards, and went to gamble, drink alcohol and occasionally for food.	Reported currently gambling on EGMs. Gambling on greyhounds and horses was his preferred gambling product.
Adrian -Male	Attended venues for food and entertainment with his supporter. Would also have lunch while there.	Did not currently gamble. Had reported gambling on EGMs at some point in his life.
Martha -Female	Attended an RSL with her husband and friends. Social outing for food and to gamble. Reported going once a month or every fortnight.	Regularly and currently gambled on EGMs and bought raffle tickets on special occasions.
Michael -Male	Reported attending venues but unclear which venue. Went with his Mum to play EGMs and for meals.	Gambled on EGMs with a parent, although unclear how frequently. Reported gambling on Melbourne Cup with family.
Bonnie -Female	Went to the sports club with family for special occasions. Attended for the food but also gambled while there.	Currently gambled on EGMs and Keno. Had gambled on sports in the past and family sweeps for Melbourne Cup.
Callum -Male	Attended pubs to socialize with friends and watch sport.	Did not currently gamble, reported using EGMs once or twice.
Ruth -Female	Reported attending a pub, and said she went all the time. Attended for the meals and to socialize.	Did not indicate that she currently gambled. Reported gambling on the Melbourne Cup and EGMs but not anymore.
Kathryn -Female	Went to an RSL weekly with her husband or on her own. Attended for food, exercise classes, to gamble, and was a member.	Indicated currently gambling on EGMs when she had the money. Had participated in raffles before.
Bethany -Female	Attended a hotel with her supporter and other residents every weekend. Socialized and went for meals while there.	Had gambled on EGMs, didn't indicate frequency.
Hazel -Female	Went to a hotel regularly with a friend. Socialized, had coffee there.	Had experienced harm from EGMs and did not currently gamble.
Oliver -Male	Reported going to the pub with family to gamble on EGMs.	Did not specify but indicated that he gambled on EGMs while with his family.

### Engagement in Gambling Activities

All participants recalled gambling in some way and at some point in their life, with 11 participants indicating that they had recently or currently gambled. People had a range of attitudes toward gambling. For example, some participants highlighted that gambling was a fun activity: “*gambling means fun*.” Others described that some forms of gambling were deceptive or had negative perceptions of gambling products. For example, one participant who had used EGMs demonstrated negative feelings toward EGMs, perceiving that many features of the machines and gambling environment made them quite dangerous:

*Once or twice, didn't really win. I can see the illusion in it though. It gives you the deception of time. It hasn't really moved when you're in there, because the lights are so bright and catchy. So you don't notice the time flying by. And have you noticed generally speaking, there are no clocks in there*—Callum, Male.

A few participants described that gambling had risks and benefits. For example, one participant, who was a regular gambler, thought gambling could be fun, but was also quick to retell experiences of harm:

*Well it can be fun, but it all depends on how you take it. You don't put too much money in, because they gobble up your money. Like some people are mad gamblers. Even one woman we saw in on New Year's Eve. She wasn't happy. She lost $600 and that's all her pension gone. And she wasn't happy—*Kathryn, Female.

Most engagement with gambling occurred with family members in community gambling venues. Two participants said they went to venues such as pubs and clubs specifically to gamble, and that they were currently going to places to gamble on their own. A few participants mentioned that they went to pubs or clubs to “*play the pokies*” or said “*I love the pokies*” but often attended for other non-gambling activities.

A few participants had engaged in gambling at wagering venues or at the casino. While some participants stated that they did not gamble on horse racing, there were some who did recall, in common with many Australians, being involved in the Melbourne Cup. This was commonly a family activity often as part of a sweep or informal bets amongst family. One participant remembered going to a betting shop with his mother for the Melbourne Cup, and picking the horses from the newspaper and “*put money on it, Mum helps me do it*.” Another participant thought that he had a better chance of winning at the TAB than on an EGM:

*TAB's alright because you've got a better chance on them. You don't press the button, you don't have to press the button or anything*—Lenard, Male.

### Interpretation of the Structural Characteristics of Gambling Products

Participants could recall the structural characteristics of different gambling products, and in particular EGMs. For example, they were able to describe what EGMs looked like and how they sounded, commenting on the “*bright colors*” or the “*noises*.” Even those participants who seemed to have a limited understanding of EGMs, were able to identify how to gamble on EGMs. For example, one participant stated, “*you've just got to put your money in and press the button*,” while another participant described that:

*We sit together and we put the money in, and we push the buttons*—Oliver, Male.

This was also the case for other gambling products that were shown to participants such as Keno, and participants noted that bingo was when “*they call the numbers out and then you say bingo*.”

A few participants named the specific design of EGM that they preferred. Preference for particular brands occurred because the EGM was a “*one or two cent machine*.” Those who named a specific type of machine also perceived that they had a better chance of winning on that particular machine—“*you always win on that one*.” One participant had said that her friend had told her to “*pick the machine that draws you in*,” while another participant stated that she looked at the machines and picked the one that she liked:

*I like the two cent Rhinos. I just look at the machine and see what it's like. If I don't like it, I don't play*—Martha, Female.

There were a few participants who indicated that they gambled regularly on EGMs. These participants were able to go into detail about how to “play” the machines. For example, when prompted they could describe technical aspects of EGMs such as “*lines*,” “*spins*,” the “*power play*,” and “*trying to get three of them in a row*.” Only a few participants were skeptical about the ability to win money on EGMs. For example, one stated that EGMs were “*all taxed now you can't win much*,” while another participant recalled that her mother had told her that EGMs were “*set to lose.”*

### Conceptualization of Gambling Harm

Most participants were able to provide a description of the negative consequences associated with gambling. Participants described that losing money was the main negative consequence associated with gambling. For example, participants stated that people could “*spend all their money*” on gambling, and that this meant that people would not be able to afford to pay bills or could “*lose their house*.” A few participants stated that having a gambling addiction could mean that people would spend too much time away from family members, and that this could lead to relationship breakdowns. One participant spoke about the potential for individuals to commit crimes, because they would “*steal things*” and “*go to jail*.” A few participants had known individuals who had had problems with gambling, and this informed their understanding of gambling related harm:

*I've got a friend she has $1,000 and she can put a whole pension on the pokies. She will say to me ‘I spent my whole pension on the pokies’ and I'll say ‘well don't you think you got a problem?’ But she says, ‘but I like them’. I said, ‘yes, I know you do, but what are you going to eat for the rest of the week, how are you going to get around for the rest of the week?’*—Martha, Female.

Another participant said:

*And I've heard stories of someone that my parents used to know. But she used to have two houses and she kind of lost the two houses. She used to just sit at that tavern and just play, play, play, play—*Callum, Male.

There were a few participants who had personally experienced gambling related harm. While these participants said that they no longer gambled, they still attended gambling venues for social activities. All talked about the financial harms they had experienced, including that they had “*wasted a bit of money*.” One participant who described her family as the initial reason she was introduced to gambling, described how her support worker had helped her to get her life back on track:

*I lost a lot. And it was bad and I couldn't pay my bills. I couldn't do this and that, and then I was up to here* [hand movement over her head] *in debt*. [name of support worker] *had to undo the debt and get me back to where I was—*Hazel, Female.

Some participants had specifically discussed the risks associated with gambling for people with intellectual disability, including that they may be vulnerable to harm because they were not fully informed of the risks associated with gambling. Specific risks included that having a disability pension meant that individuals felt they had disposable income to spend on gambling, and that those who accompany people with intellectual disability to venues may (un)intentionally encourage gambling:

*More people with intellectual disabilities and myself, are with their parents or with their support workers. And their support workers or their parents encourage them to* [gamble]—Leah, Female.

### Understanding of and Engagement With Gambling Harm Reduction and Help Seeking Strategies

Some participants had a strong idea of what people should do if they were experiencing gambling related harm. A few participants could name formal support services such as “*Gamblers Help*,” or suggested people should call the “*gambling hotline*.” Other participants identified that it was important for individuals to “*talk to someone and seek help*.” While most participants were unable to specifically describe who they would talk to, or how someone could seek help, there was still an implicit recognition that support was available for people who were experiencing a problem with gambling:

*Oh, they should tell them to go see someone. And work it out instead of spending all their money—*Ruth, Female, 70 years.

While participants expressed concern for people who were experiencing gambling harm, very few stated that they would say something about someone else's gambling if they were worried about them. Participants stated that they would be worried they would upset the person, would receive a negative reaction, or did not believe they could do anything to address the persons behavior:

*You can't really say anything to them. Because if you say something to them they are likely to say “mind your own business.” I think they have to find out the hard way. But I say not to put too much money in, but people have to find out for themselves…really—*Kathryn, Female.

Less than half of participants recognized the term ‘responsible gambling.’ Those who had, had seen these messages on EGMs, in the bathrooms at pubs and clubs, and on television commercials. For example, one participant said:

*Yeah all over the RSL there is always* [one] *on all the machines. It says “gamble responsibly*.”—Martha, Female.

Participants interpreted the responsible gambling message to mean to “*set limits*,” “*don't gamble all of your money*” and “*you have to be safe*.” One participant had a specific example of how to be responsible while using EGMs:

*To me it's like you got to be responsible of how long you are on a pokie for. You got to make sure you've got the right amount of money. And when you run out of money, you know you got to finish. Because most people aren't responsible. Because they just keep feeding it in. My fear is if you keep feeding it you're not going to win—*Bonnie, Female.

Most participants had not heard of the term ‘responsible gambling,’ with some stating that they wished that there were warning messages about gambling in venues and EGMs. One woman who had experienced gambling harm stated that she did not recall any messaging or interventions from staff members when she was gambling.

Many of the participants had strong personal responsibility narratives about ways to minimize the harms associated with gambling. Common themes included setting limits, being in control of gambling behaviors, not spending more than people could afford, and were fearful of becoming addicted to gambling. One participant described how her mother's gambling had led her to perceive that her own gambling behaviors were responsible:

*My mother is a gambler, but I don't talk to my mother now. My family, because my mother gambled so much, we were living out of suitcases because we didn't know where we were going to live next. So that was very, very hard, on me. So, I set myself a limit and it is the same, if I drink too much, I will tell my husband I've drunk too much. And I know my limits when I stop gambling and when I stop drinking—*Martha, Female.

## Discussion

This study aimed to understand the factors that may influence how and why people with intellectual disabilities may engage in, and understand the harms associated with gambling. The findings from the study raise three points for discussion.

First, participants engaged with a range of gambling products in community-based settings and had a high level of awareness of and receptivity toward gambling. The product that they engaged with the most in this study were EGMs. Further research should seek to systematically examine the different types of gambling products that people with intellectual disabilities engage with and prefer. While this study focused on people who attended community gambling venues where EGMs are one of the main forms of gambling, there were a few factors that raise particular concern about participant engagement with EGMs. EGMs are a high intensive gambling product, and are broadly considered to be the most harmful form of gambling in Australia (5, 43, 44). While some participants in this study recognized the harms associated with these machines, others were highly receptive to gambling on EGMs. This included having specific branded machines that they preferred playing and being able to describe some of the structural characteristics of the machines that they particularly valued. They also had significant misperceptions about the machines, including the likelihood of winning, and that some machines were more likely to be “lucky” or “pay out” as compared to others. Research has demonstrated that misperceptions about the structural characteristics of EGMs, and in particular the likelihood of winning, is a risk factor for gambling harm (43, 45). While further research is needed to understand these factors in more detail, the findings from this study indicate that population based harm prevention strategies are not reaching those who may be significantly vulnerable to engaging with, and experiencing harm from, EGMs.

Second, participants engagement with gambling was often facilitated by individuals in their immediate formal and informal social networks. Research both in gambling and other areas of public health have demonstrated the role of social networks, and particularly family members in risky health behaviors (46). Research has also shown that community gambling venues may on the one hand, provide supportive and inclusive environments for individuals who may otherwise feel marginalized or excluded from society, but on the other hand, contain products that may cause these individuals significant harm (32, 47). Practical responses to these issues should not only consider the experiences and views of people with intellectual disability, but also their families and supporters in programs aimed at creating awareness about the risks associated with these venues, and in providing alternative recreational spaces.

Third, while participants in this study understood that gambling had a range of negative consequences associated with it, many understood gambling related harm as an issue associated with personal responsibility. It is noteworthy that the harm minimization messages that were recalled by participants were all based on written messages that they had seen in venues and on television. Given the potential limited literacy levels of people with intellectual disability, this could be a reason why more than half said that they had not seen a harm minimization message, with some stating that warning messages were needed. Current gambling harm minimization campaigns in Australia are often focused on personal responsibility messages (48), however research shows that this messaging is largely ineffective and in some instances has been suggested to increase shame and stigma for individuals (48). While a public health approach for reducing gambling harm calls on a broader approach than education and messaging, there is a need for responsibility campaigns to shift the onus off individual behaviors and consider the broader range of factors that are contributing to harm. However, given the retention of people with intellectual disability to current campaigns, it highlights an obvious need for the development of targeted evidence based campaigns inclusive of people with intellectual disability and that are effective in reducing gambling harm. This could explore innovative ways of delivering harm prevention messages in venues, such as the use of independently developed messages which are audio-played at regular intervals in gambling venues.

### Limitations

There are three limitations to this study. First this study specifically recruited individuals who attended community gambling venues which all contain EGMs. While most participants in this study had gambled on EGMs, this may not be generalisable to the gambling behaviors of all people with intellectual disability. However, it does highlight that those who attend these venues may engage with EGMs, with some reporting experiencing significant harm from these machines. Second, it was difficult to gauge participants' frequency of gambling behaviors, or the amount of time and money they spent on particular gambling products. Future research should explore the most appropriate methods for capturing the frequency with which people with intellectual disability engage in different forms of gambling, as well as methodologies for appropriately understanding the harm that they may experience from gambling. Finally, there may have been some elements of social desirability, which may have resulted in participants either inflating or underestimating their gambling behavior and knowledge depending on how they perceived the positive or negative impacts of gambling.

## Conclusion

This study demonstrated the range of potential factors influencing people with intellectual disability's gambling attitudes and behaviors. It highlights the need for researchers, practitioners and policy makers to consider the impact of gambling on the lives of people with intellectual disability. This includes understanding how people with intellectual disability interact with, and understand gambling products, the range of determinants that may contribute to their engagement with gambling products and environments, and the range of policy and practice responses aimed at preventing gambling harm in this population subgroup. Future research should seek to comprehensively explore the complex range of factors that may contribute to the intersection of social conditions for experiences of disability, gambling and harm.

## Data Availability Statement

The datasets for this article are not publicly available because: we need to ensure the participants are kept confidential. Requests to access the datasets should be directed to Hannah Pitt, hannah.pitt@deakin.edu.au.

## Ethics Statement

The studies involving human participants were reviewed and approved by Deakin University Human Research Ethics Committee. The patients/participants provided their written informed consent to participate in this study.

## Author Contributions

HP: Contributed to study design, data collection, data analysis, and preparing the manuscript. ST: Contributed to the design of the study, data collection and interpretation, and the critical revisions of the manuscript. JW: Contributed to the design of the study, data collection, and the critical revisions of the manuscript. RS: Contributed to the design of the study and the critical revisions of the manuscript. KM: Contributed to the design of the study and the critical revisions of the manuscript. SB: Contributed to the design of the study and the critical revisions of the manuscript. All authors have read and approved the manuscript.

## Conflict of Interest

The authors declare that the research was conducted in the absence of any commercial or financial relationships that could be construed as a potential conflict of interest.
